# How gut bacteria influence the brain barriers, neuroinflammation and Alzheimer’s disease: a role for bacterial metabolites and extracellular vesicles

**DOI:** 10.1002/alz.083658

**Published:** 2025-01-03

**Authors:** Roosmarijn E Vandenbroucke, Barriers in Inflammation lab, VIB‐UGent Center for Inflammation Research, Ghent, Belgium

**Affiliations:** ^1^ VIB‐UGent Center for Inflammation Research, Ghent Belgium

## Abstract

**Background:**

The brain is shielded from the peripheral circulation by central nervous system (CNS) barriers, comprising the well‐known blood‐brain barrier (BBB) and the less recognized blood‐cerebrospinal fluid (CSF) barrier located within the brain ventricles. The gut microbiota represents a diverse and dynamic population of microorganisms that can influence the health of the host, including the development of neurological disorders like Alzheimer’s disease (AD). However, the intricate mechanisms governing the interplay between the gut and brain remain elusive, and the means by which gut‐derived signals traverse the CNS barriers remain unclear.

**Methods:**

Microbiota was removed via antibiotics treatment or using germfree mice. *App* ^NL‐G‐F^ mice were used as mouse model for AD. *Helicobacter pylori* (*H. pylori*) and total microbiota‐derived extracellular vesicles (EVs) were purified using size exclusion chromatography (SEC) and density gradient, followed by quality control. Bacterial‐derived metabolites and EVs were administered to mice via oral gavage. Brain barrier tightness was analyzed using FITC‐dextran leakage, tight junction visualisation and immune cell infiltration in the brain. AD pathology was determined via Aβ plaque load (ELISA & plaque immunostainings) and cognitive behavioural tests. Neuroinflammation was determined via analysis of glial activation, qPCR, cytokine analysis and immune cell infiltration into the brain.

**Result:**

Our research uncovers the essential role of gut microbiota in the formation and maintenance of tight CNS barriers. Notably, certain metabolites reinforce CNS barrier integrity, irrespective of vagal nerve influence, safeguarding against AD‐related deterioration of brain integrity. Additionally, specific pathogens like *H. pylori* generate EVs, so called outer membrane vesicles (OMVs) that trigger brain inflammation, accelerating AD‐associated neuropathological processes, depending on complement 3 (C3) signaling. Interestingly, utilizing germ‐free *App* ^NL‐G‐F^ mice, we observed that also oral administration of gut microbiota‐derived bacterial EVs (bEVs) exacerbate Aβ pathology and disrupt astrocyte‐microglia‐neuron networks.

**Conclusion:**

In summary, the influence of bacteria‐derived metabolites and EVs on brain barriers and AD pathogenesis underscores the significance of unraveling these mechanisms. Such understanding offers fresh insights, potentially guiding the development of innovative therapeutic strategies for neurological disorders.

